# Sensor-based assessment of fertilizer strategies in soybean: linking SPAD, NDVI, plant height, and thermal imaging with biomass accumulation

**DOI:** 10.1186/s12870-025-07628-x

**Published:** 2025-11-24

**Authors:** Süreyya Betül Rufaioğlu, Murat Tunç

**Affiliations:** 1 Department of Soil Science and Plant Nutrition, Agriculture Faculty, Harran University, Sanliurfa, Türkiye; 2 Department of Field Crops, Agriculture Faculty, Harran University, Sanliurfa, Türkiye

**Keywords:** Soybean, Optical sensor, SPAD, NDVI, Thermal imaging, Fertilizer management, Precision agriculture

## Abstract

This study aimed to investigate the temporal effects of different fertilization strategies on the physiological, morphological, and biomass-related traits of soybean under controlled greenhouse conditions. Individual and combined applications of urea, zinc (Zn), and microbial inoculants were evaluated using a multi-sensor approach. Optical parameters (SPAD, NDVI), plant height, and thermal imaging were monitored across days after onset (DAO**)**, and post-harvest biomass traits were measured to establish integrative relationships. The findings demonstrated that SPAD values increased by 18–27% and NDVI by up to 22%, with combined treatments (Urea + Microbial, Zn + Microbial) showing the most pronounced improvements. Urea + Microbial increased plant height by 15% and fresh biomass by 28% compared to the control. Thermal imaging revealed a 1.8–2.5 °C reduction in canopy temperature under combined treatments, indicating enhanced stomatal regulation and water-use efficiency. Strong positive correlations (*r* = 0.71–0.84) between SPAD/NDVI and post-harvest biomass confirmed the reliability of early-stage sensor measurements for predicting yield-related traits. Importantly, the integration of microbial inoculants with mineral fertilizers enhanced both physiological resilience and water-use efficiency, while the identification of tentative threshold values for SPAD (~ 35) and NDVI (~ 0.60) provides practical benchmarks for fertilization decisions and automation in precision agriculture. Overall, this study highlights the utility of combining optical and thermal sensing with morphological and biomass assessments to optimize fertilization strategies in soybeans. The results provide novel insights into the role of micronutrient (Zn) and microbial management in crop monitoring and underline the potential of sensor-based approaches to improve nutrient efficiency and support sustainable agricultural production.

## Introduction

Soybean (*Glycine max* L.) is a fundamental legume of strategic importance for both food and feed industries worldwide. Its widespread use as a global source of oil and protein, coupled with the rising population and food demand, necessitates improvements in production efficiency [1–4]. One of the major challenges in soybean production is the effective management of nutrients, particularly nitrogen. Excessive fertilizer uses leads to economic losses and pollution in soil and water ecosystems, while insufficient fertilization results in yield reductions [5, 6]. Therefore, in recent years, agricultural research has increasingly focused on developing methods that enhance productivity while ensuring environmental sustainability through precision agriculture practices [7]. In this context, sensor technologies have gained growing importance as rapid and non-destructive tools for monitoring plant physiology. The leaf chlorophyll meter (SPAD), a widely used optical sensor, provides a reliable indication of nitrogen status and photosynthetic capacity, and is considered an important decision-support tool in nutrient management, particularly in soybean cultivation [8, 9]. The response of SPAD values to fertilizer applications and growth stages enables effective monitoring of temporal changes in chlorophyll concentration [10]. Similarly, vegetation indices, particularly NDVI, allow quantitative assessments of plant growth by reflecting leaf area index, canopy vigor, and photosynthetic activity [11, 12]. Particularly in drought- and heat-sensitive crops, thermal imaging enables the early detection of stress conditions and provides valuable insights when combined with optical indices [13, 14]. The temporal variation of NDVI has been widely used to evaluate the effects of different fertilizer strategies and cultivars on growth dynamics [15, 16]. However, parameters derived solely from optical sensors may be insufficient to fully explain the physiological responses of plants. At this point, thermal imaging serves as a complementary method, as canopy temperature is closely associated with water status, stomatal conductance, and transpiration [17, 18]. Since water availability, along with nitrogen, is one of the most critical factors for soybean productivity, integrating thermal data with fertilizer management offers new opportunities for improved stress detection and resource-use efficiency [19, 20]. Nevertheless, most sensor-based studies have predominantly focused on macronutrients (N, P, K). Research addressing the combined effects of micronutrients, particularly zinc (Zn), and microbial inoculants on physiological and morphological traits through sensor-based monitoring remains limited. Zn plays a regulatory role in chlorophyll synthesis, enzyme activation, and root development, while its deficiency restricts photosynthetic efficiency and biomass accumulation [5, 21, 22].Microbial inoculants, on the other hand, enhance nutrient mineralization, strengthen root–soil interactions, and improve both water- and nutrient-use efficiency, thereby facilitating plant adaptation under stress conditions [23]. Recent evidence further highlights that Zn-solubilizing and mineral-dissolving bacteria contribute significantly to nutrient availability and crop resilience under stress. Therefore, the scientific gap lies not in the technical features of the sensors themselves but in their potential to capture physiological responses driven by micronutrient and microbial fertilizer applications. In this context, the joint evaluation of optical and thermal sensors with morphological traits and post-harvest biomass parameters provides a holistic framework for fertilizer management and yield estimation.

This study aims to address this gap. The individual and combined applications of urea, Zn, and microbial inoculants were evaluated in soybean with respect to physiological, morphological, and biomass traits using SPAD, NDVI, thermal imaging, and plant height measurements. Furthermore, these sensor-based parameters were validated against post-harvest biomass data to test their accuracy. In this way, the study provides novel contributions to precision agriculture by demonstrating the potential of sensors to monitor physiological responses associated with micronutrient and microbial fertilization strategies.

## Materials and methods

### Plant material

In this study, the soybean cultivar Gapsoy-16, which is widely cultivated in the region and developed by the GAP Agricultural Research Institute, was used as the plant material. The plant height of this cultivar ranges from 99 to 137 cm, and the upper surface of young leaves exhibits a dark copper coloration. Prior to sowing, the seeds were subjected to surface sterilization, and subsequently, seeds of uniform size were selected to ensure germination uniformity. An equal number of seeds were sown in each pot, and after emergence, thinning was performed to maintain only one plant per pot.

### Pot experiment design and treatments

The experiment was initiated on May 1, 2025, with the sowing of soybean seeds under semi-controlled greenhouse conditions and was completed on July 9, 2025. The arrangement of pots in the greenhouse followed a randomized complete block design (RCBD), consisting of 7 treatment levels with 4 replications each, for a total of 28 pots. The study was conducted in the semi-controlled research greenhouse of Harran University, Faculty of Agriculture. The irrigation program was applied uniformly across all treatment groups. Each pot was filled with 1 L of field soil. As a basal fertilizer, DAP (Diammonium Phosphate, 18–46-0) was applied to each pot at the time of sowing. The microbial fertilizer used in the study, containing the strain Clonostachys rosea st1140, was developed and produced by the GAP Agricultural Research Center. The selection of seven fertilizer treatments in this study was based on their agronomic relevance and complementary roles in soybean growth. Urea was included as the primary nitrogen source, as nitrogen is essential for chlorophyll biosynthesis, protein formation, and canopy development [24, 25]. Zinc (Zn) was selected because of its regulatory functions in photosynthetic enzymes, auxin metabolism, and root development, which directly influence stress tolerance and yield [26]. The microbial inoculant was applied to evaluate the contribution of beneficial microorganisms to nutrient mineralization, soil–root interactions, and enhanced water- and nutrient-use efficiency [27, 28]. The combined treatments (Urea + Zn, Urea + Microbial, Zn + Microbial) were designed to assess the synergistic effects of mineral and biological fertilizers, considering that microbial inoculants can improve nutrient availability and mitigate stress, while mineral fertilizers provide immediate nutrient supply. Previous studies demonstrated that such integrations enhance soybean growth, chlorophyll concentration, and biomass accumulation more effectively than single applications [29–31]. Therefore, these seven treatments provided a comprehensive framework to compare individual and integrated fertilization strategies in soybean under controlled conditions. All physiological, morphological, and biomass parameters were evaluated based on the number of days after onset (DAO), where DAO refers to the days counted from the initial plant onset. The details of the treatments and fertilizer compositions are presented in Table 1.

**Table 1:** Description of Foliar Fertilizer Treatments and Application Doses

Treatment	Abbreviation	Content	Fertilizer Dose
Control	–	No fertilizer applied	–
Urea	Urea	Urea (46% N) – primary nitrogen source for chlorophyll biosynthesis, protein formation, and canopy development	10 kg/da
Zinc sulphate	Zn	ZnSO₄·7H₂O (22% Zn) – supports photosynthetic enzymes, auxin metabolism, and root development	2.5 kg/da
Microbial fertilizer	Microbial	*Clonostachys rosea* st1140 strain – enhances nutrient mineralization, soil–root interactions, and water/nutrient-use efficiency	1 L/da
Urea + Microbial	Urea + Microbial	Combination of Urea (46% N) and *Clonostachys rosea* st1140	10 kg/da + 1 L/da
Zinc sulphate + Microbial	Zn + Microbial	Combination of ZnSO₄·7H₂O (22% Zn) and *Clonostachys rosea* st1140	2.5 kg/da + 1 L/da
Urea + Zinc sulphate	Urea + Zn	Combination of Urea (46% N) and ZnSO₄·7H₂O (22% Zn)	10 kg/da + 2.5 kg/da

### Optic sensor measurements

Physiological observations were conducted at weekly intervals, and all measurements were taken on the same day. Measurements were based on four replications, and the data obtained from each replication were averaged and used in the statistical analyses. All sensor measurements were carried out consecutively between 13:00 and 15:00 to minimize diurnal variation. Leaf chlorophyll content was determined using a SPAD-502 Plus (Konica Minolta Inc., Osaka, Japan). The SPAD is a non-imaging optical sensor that requires direct leaf contact; measurements were performed by placing the middle canopy leaves into the clamp of the device, and four readings per replication were averaged. The SPAD meter does not directly measure the absolute chlorophyll concentration but provides a relative chlorophyll content index (CCI) derived from the difference in light absorption at red (650 nm) and near-infrared (940 nm) wavelengths. This index is widely used as an indirect indicator of leaf nitrogen status in plant nutrition studies [25, 32]. The Normalized Difference Vegetation Index (NDVI) was measured using a Trimble GreenSeeker Handheld Crop Sensor (Trimble Inc., Sunnyvale, CA, USA). NDVI measurements were conducted on the same days as SPAD readings, with the sensor positioned at a fixed distance of approximately 70 cm above the canopy of each pot. Four replicate measurements per treatment were taken and averaged. The GreenSeeker calculates NDVI based on the ratio of reflectance between red (656 nm) and near-infrared (774 nm) wavelengths, allowing the assessment of plant biomass, photosynthetic capacity, and stress status [33–35].

### Morphological measurement

Plant height was measured using a ruler by recording the distance from the soil surface to the highest point of the main stem, a standard method frequently employed in agronomic studies to quantify structural growth [24, 36]. Measurements were conducted weekly on the same days as SPAD, NDVI, and thermal observations, between 13:00 and 15:00. In each treatment, plant height was recorded from four replicated plants (*n* = 4), and the average value was used for statistical analysis. Measurements were performed at five observation times corresponding to 6, 14, 20, 26, and 33 days after sowing.

### Thermal image processing and analysis

**Fig. 1:**
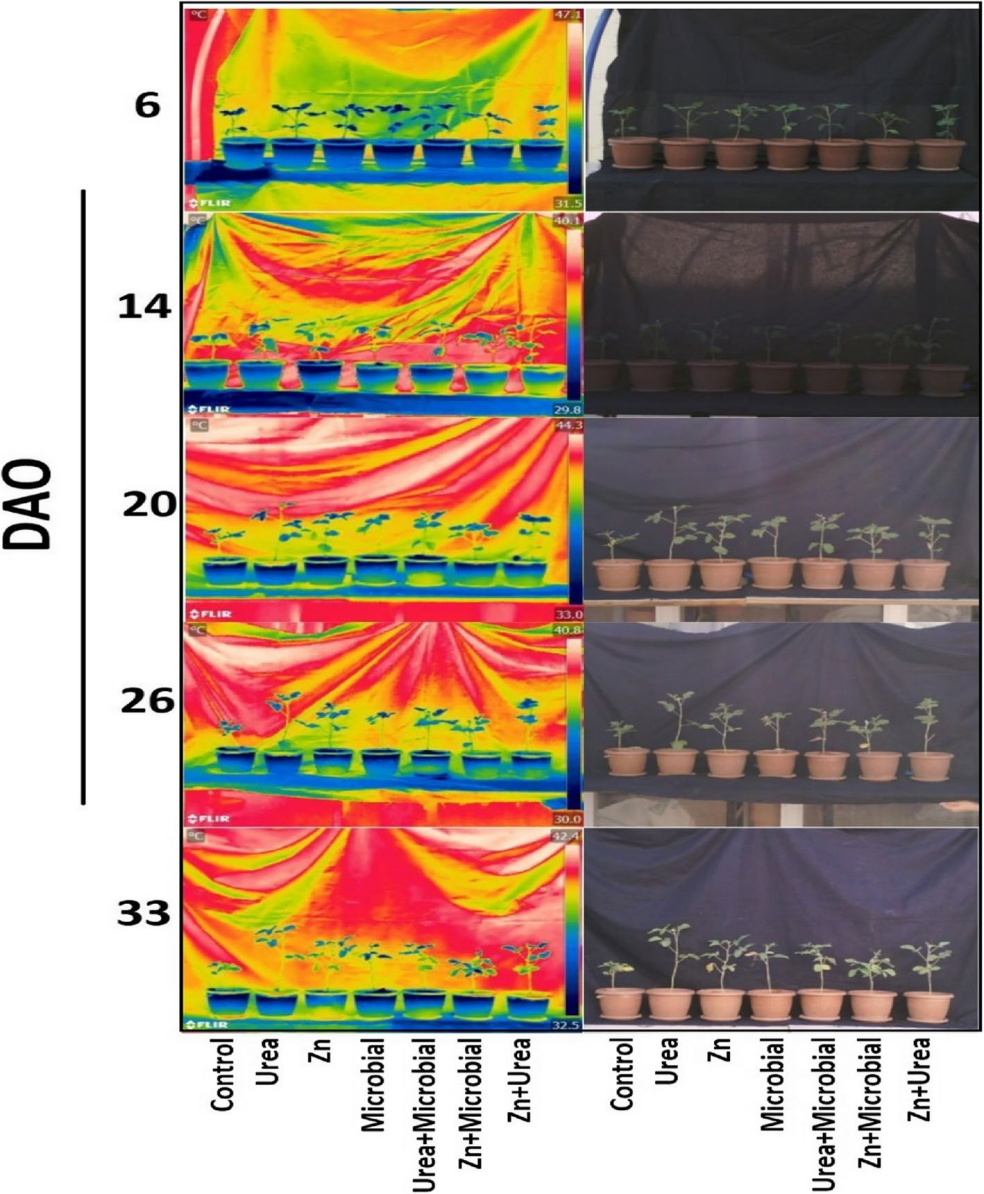
Thermal and visual images of soybean plants under different treatments (Control, Urea, Zn, Microbial, Urea + Microbial, Zn + Microbial, Urea + Zn) taken at different days after onset (DAO: 6, 14, 20, 26, 33). Thermal imaging was performed with FLIR camera to assess canopy temperature, while visual images show morphological development among treatments

Thermal imaging studies were carried out using a thermal infrared camera, FLIR T540 (FLIR Systems Inc., Wilsonville, USA). The device is equipped with a detector offering a resolution of 464 × 348 pixels and a thermal sensitivity of < 0.03 °C (30 mK, < 30 °C), enabling the detection of even very small temperature differences. During image acquisition, a black curtain was placed as a background to eliminate external reflections and improve the accuracy of canopy temperature readings. All measurements were conducted under natural daylight conditions between 13:00 and 15:00, in order to minimize diurnal variation in leaf temperature. The camera-to-plant distance was maintained at approximately 3–4 m, and emissivity was set to 0.95, which is standard for vegetation. The camera was operated in automatic focus mode, with regular calibration checks prior to each measurement. Thermal images were processed using FLIR Tools software (version 6.4). For each potted plant, four spot measurements were taken from different canopy regions, and the average value was used to represent canopy temperature. This procedure was repeated for all pots across the seven fertilization treatments, ensuring consistent measurement across replications. The software enabled detailed pixel-based analysis of temperature distributions and allowed reliable determination of canopy and overall leaf surface temperatures for each plant. A color scale was used in the thermal images to visually assess differences among treatments (Fig. 1).

### Post-harvest parameters

Following harvest, the biomass components of the plants were thoroughly evaluated. First, the aboveground parts of each plant were cut and the fresh weight (FW) was measured using a high-precision electronic balance. The same samples were then dried at 65 °C for 48 h in a forced-air oven until a constant weight was achieved. The drying process was carried out in a Nüve FN 500 forced-air oven (Nüve, Ankara, Türkiye), after which the samples were weighed again to record the dry weight (DW) values. Following drying, moisture content (MC, %) was calculated as the ratio of the difference between FW and DW to FW; dry matter content (DMC, %) was determined as the ratio of DW to FW; the water-to-dry matter ratio (H₂O/DM) was calculated as the ratio of water content to dry matter; and water content (WC) was expressed as the absolute difference between FW and DW. These calculations were based on standard methodologies for biomass determination through fresh and dry weight measurements [37–39].

### Statistical analysis and data visualization

For the analysis of the dataset, a Completely Randomized Design (CRD) was adopted. Prior to analysis, the data were tested for normality and homogeneity of variances. Normality was assessed using the Shapiro–Wilk test, while homogeneity of variances was evaluated with Levene’s test; the results confirmed that the basic assumptions of ANOVA were met. A one-way ANOVA model was established to test the differences among treatments [40]. When statistically significant differences were detected (95% confidence level, *p* < 0.05), pairwise comparisons among groups were conducted using Tukey’s Honest Significant Difference (HSD) test. Replications used in the experiment were considered in estimating the error variance and were reported in the ANOVA table together with the relevant degrees of freedom. In addition, Pearson’s correlation coefficients were calculated to determine the relationships among the investigated traits. All statistical analyses were performed using JMP Pro 17 statistical software. Data visualization was carried out in a Python 3.10 environment. The dataset was prepared and uploaded to Google Colab in CSV format. For graphical representation, the pandas [41], matplotlib [42], seaborn [43], and scipy [44] libraries were employed. Line graphs with error bars were generated to illustrate the temporal changes of physiological parameters, while bar charts indicating statistical differences were prepared for post-harvest biomass data. Furthermore, heatmaps were constructed for correlation analyses, and biplot and loading plots were generated for principal component analysis (PCA). These visualizations not only supported the statistical results but also provided clearer insights into the interrelationships among parameters.

## Results

### Descriptive statistical analyses

The results of the variance analysis revealed that the treatments had significant effects on the physiological and yield parameters of soybean plants. Plant height and SPAD values exhibited highly significant differences across all observation periods (6, 14, 20, 26, and 33 DAO), with the highest variation observed on day 26 (*p* < 0.01). NDVI values did not differ significantly at 6 DAO (*p* > 0.05); however, they displayed statistically significant differences at 14, 20, and 26 DAO (*p* < 0.01), indicating that the treatments markedly influenced photosynthetic activity particularly during the pre-flowering stage. In terms of canopy temperature, significant differences were observed at 6 DAO (*p *< 0.01) and 14 DAO (*p* < 0.05), but no significant variation was detected at later stages (*p* > 0.05). For post-harvest traits, fresh weight was found to be strongly affected by the treatments (*p* < 0.01), whereas dry weight showed moderately significant differences (*p* < 0.05). Water content also exhibited significant variation among treatments (*p* < 0.01), while moisture content, dry matter content, and H₂O/DM ratio did not differ significantly (*p* > 0.05). Overall, the findings demonstrated that fertilizer and microbial applications exerted strong influences on vegetative growth, chlorophyll content, photosynthetic capacity, and biomass accumulation during the post-sowing growth period (Table 2).

**Table 2:** Analysis of variance (F values) for physiological parameters (plant height, SPAD, NDVI, and plant temperature) measured at different growth stages (6, 14, 20, 26, and 33 days after sowing) and for post-harvest traits (fresh and dry weight) of soybean under different treatments

Parameters	DAO (*F *Value)				
	**6**	**14**	**20**	**26**	**33**
Plant Height (cm)	24,59**	57,57**	42,73**	138,77**	97,27**
SPAD	29,28**	23,00**	42,73**	138,77**	97,27**
NDVI	1,01ns	13,75**	12,93**	24,55**	9,26**
Plant Temperature (℃)	9,90**	3,02	2,19	2,55	2,40
	Post Harvest (***F*** Value)				
Plant Fresh Weight (g/plant)	8,10**				
Plant Dry Weight (g/plant)	6,63*				
Water Content (g/plant)	5,70**				
Moisture Content (%)	0,74				
Dry Matter Content (%)	0,74				
H_2_O/DM	0,88				

^*^p < 0.05

^**^p < *0.01*)

### Findings of post-harvest observation

**Fig. 2:**
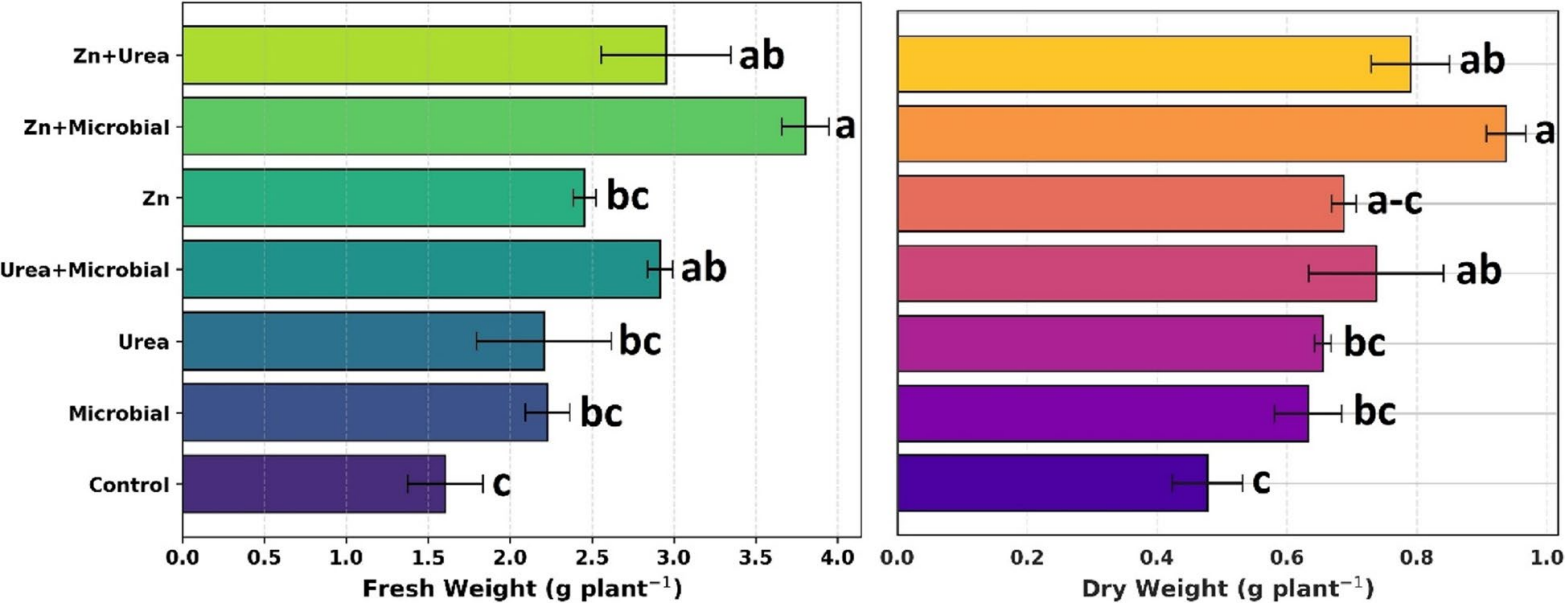
Effects of different treatments (Control, Urea, Zn, Microbial, Urea + Microbial, Zn + Microbial, Urea + Zn) on fresh weight and dry weight of soybean at harvest. Error bars represent the standard error of each treatment (*n* = 4). Different letters above the bars indicate significant differences among treatments (*p* < 0.05). Bars without letters denote non-significant differences (*p* < 0.05)

Post-harvest measurements revealed that the treatments produced significant differences in both fresh (*p* < 0.01) and dry weights (*p* < 0.05). In terms of fresh weight, the highest value was recorded under the Zn + microbial treatment, followed by the Zn + urea and urea + microbial combinations. Single applications (Zn, urea, microbial) resulted in intermediate values, while the control group exhibited the lowest fresh weight. A similar trend was observed for dry weight, where Zn + microbial and Zn + urea treatments achieved the highest dry matter accumulation, with the urea + microbial treatment showing comparable values. Once again, the control group produced the lowest dry weight. These findings indicate that the combined application of mineral fertilizers with microbial inoculants substantially enhances biomass accumulation in soybean (Fig. 2).

**Fig. 3:**
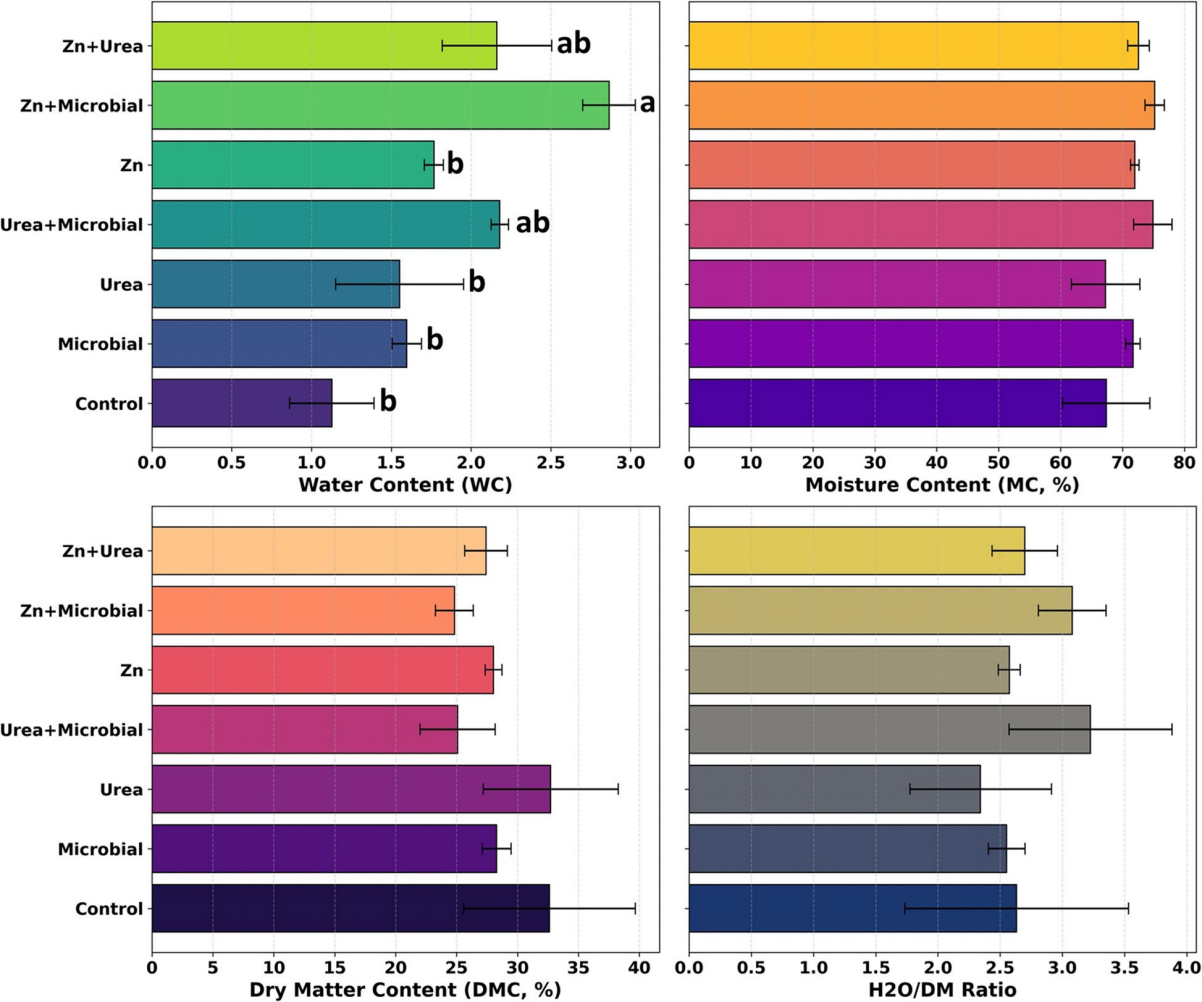
Effects of different fertilizer applications (Control, Microbial, Urea, Zn, Urea + Microbial, Zn + Microbial, Zn + Urea) on water content (WC), moisture content (MC, %), dry matter content (DMC, %), and H2O/DM ratio in soybean. Bars represent standard error values. Means followed by the same letter are not significantly different according to the statistical test (*p* < 0.05)

In soybean, the effects of different fertilizer applications on water relations revealed that the Zn + Microbial and Zn + Urea treatments provided the highest values in terms of water content (WC), which differed significantly among treatments (*p* < 0.01), while the control group exhibited the lowest levels. For moisture content (MC), no statistically significant differences were observed (*p* > 0.05) among treatments, and all remained at similar levels. Regarding dry matter content (DMC), although urea application resulted in relatively higher values compared to the other treatments, the differences were not statistically significant (*p* > 0.05). In the H₂O/DM ratio, the Urea + Microbial and Zn + Microbial combinations were prominent, whereas the control and microbial treatments remained at lower levels; however, these variations were not statistically significant (*p* > 0.05). These findings indicate that the application of zinc and urea, either alone or in combination with microbial fertilizers, can enhance the water retention capacity of the plant and thereby exert a positive influence on physiological performance (Fig. 3).

**Fig. 4:**
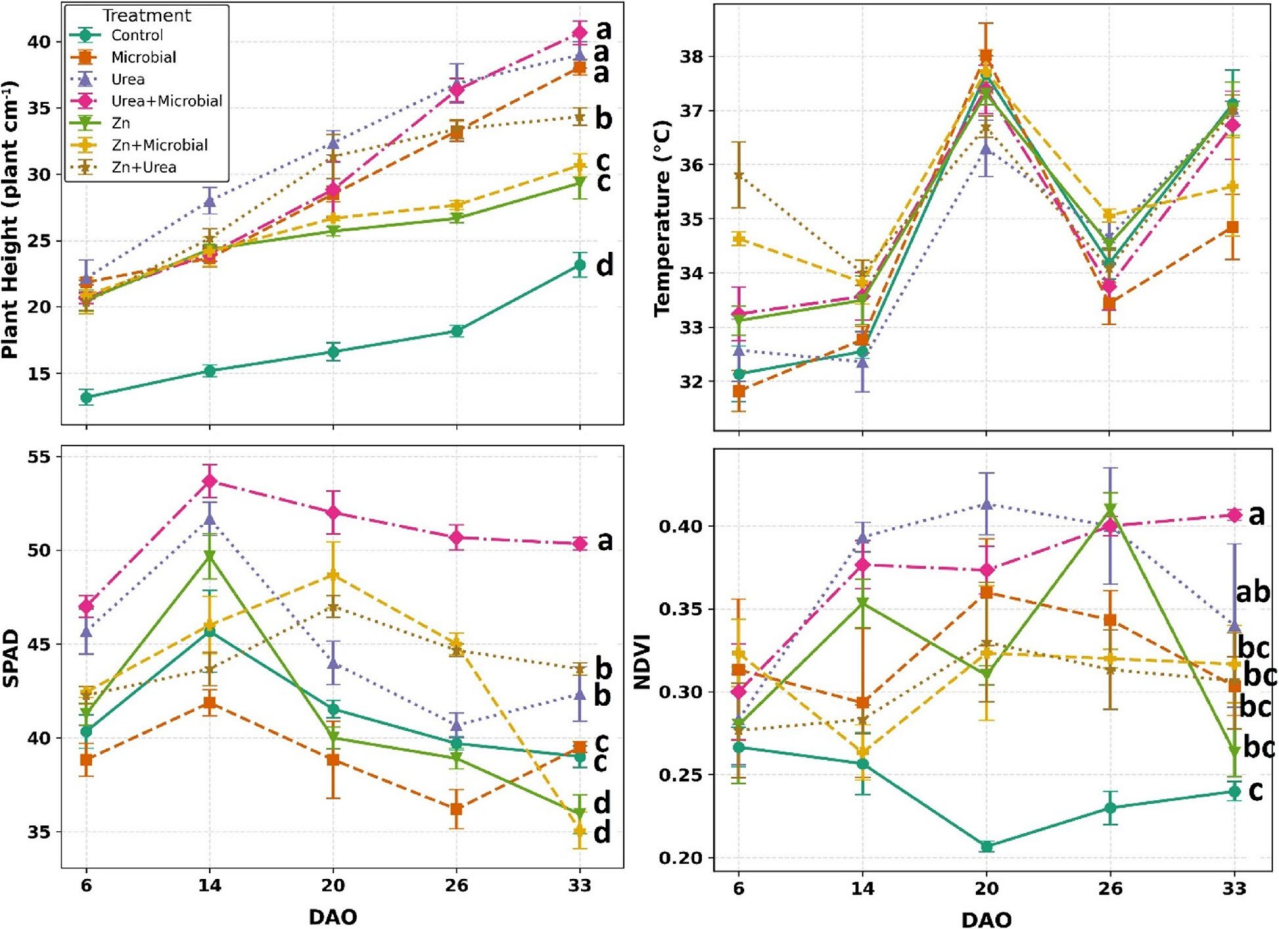
Plant height, canopy temperature, SPAD, and NDVI of plants under different treatments. Error bars represent the standard error of each treatment (*n* = 4). Different letters above the bars indicate significant differences among treatments (*p* < 0.05). Bars without letters denote non-significant differences (*p* < 0.05)

Temporal analyses demonstrated that the treatments had pronounced effects on plant height, leaf temperature, SPAD, and NDVI values. Plant height consistently increased across all measurement periods, with statistically significant differences observed at each stage (*p* < 0.01). The urea + microbial treatment reached the highest value on day 33, while the control group consistently exhibited the lowest height. SPAD values peaked on day 14, with the urea + microbial treatment achieving the highest chlorophyll index; in contrast, the control and single applications remained at lower levels, and the differences were highly significant (*p* < 0.01). NDVI results revealed no significant variation at 6 DAO (*p* > 0.05), but statistically significant differences were recorded at 14, 20, 26, and 33 DAO (*p* < 0.01). At 20 and 26 DAO in particular, the urea + microbial and Zn + microbial combinations reached the highest vegetation index values, whereas the control group lagged behind all other treatments. Overall, these findings indicate that the combined application of mineral and microbial fertilizers substantially improved growth, chlorophyll concentration, and photosynthetic capacity in soybean plants (Fig. 4).

Thermal analyses further supported the effects of fertilizer treatments on canopy temperature dynamics. At 6 DAO, significant differences were observed among treatments (*p* < 0.01), with the control group exhibiting relatively higher temperatures compared to the combined applications. At 14 DAO, differences remained significant but at a lower level (*p* < 0.05), again showing that the combined treatments (particularly Urea + Microbial and Zn + Microbial) maintained cooler canopy profiles. In contrast, no statistically significant differences were detected at 20, 26, and 33 DAO (*p* > 0.05), although combined treatments consistently displayed slightly lower canopy temperatures compared to the control group. These results indicate that mineral and microbial fertilizers, when applied together, help reduce canopy temperature and mitigate stress during the early growth stages (Table 3).

**Table 3:** Mean canopy temperature (°C ± SE) of soybean plants under different fertilizer treatments at five growth stages (6, 14, 20, 26, and 33 days after sowing, DAO)

Treatment	6 DAO (°C)	14 DAO (°C)	20 DAO (°C)	26 DAO (°C)	33 DAO (°C)
Control	32.13 ± 0.51^c^	32.55 ± 0.13	37.67 ± 0.34	34.17 ± 0.29	37.12 ± 0.62
Urea	32.57 ± 0.57^bc^	32.36 ± 0.56	36.30 ± 0.52	34.66 ± 0.51	37.03 ± 0.14
Zn	33.11 ± 0.27^bc^	33.49 ± 0.45	37.31 ± 0.21	34.52 ± 0.10	37.03 ± 0.49
Microbial	31.82 ± 0.38^c^	32.76 ± 0.25	38.02 ± 0.59	33.44 ± 0.38	34.84 ± 0.60
Urea + Microbial	33.24 ± 0.50^bc^	33.56 ± 0.44	37.38 ± 0.45	33.75 ± 0.43	36.72 ± 0.63
Zn + Microbial	34.63 ± 0.13^ab^	33.83 ± 0.40	37.71 ± 0.40	35.05 ± 0.12	35.60 ± 0.92
Zn + Urea	35.81 ± 0.61^a^	34.00 ± 0.24	36.70 ± 0.20	34.08 ± 0.37	36.99 ± 0.29

Mean values within the same column followed by different letters indicate statistically significant differences according to the multiple comparison test (*p* < 0.05)

Values without letters are not significantly different

**Fig. 5:**
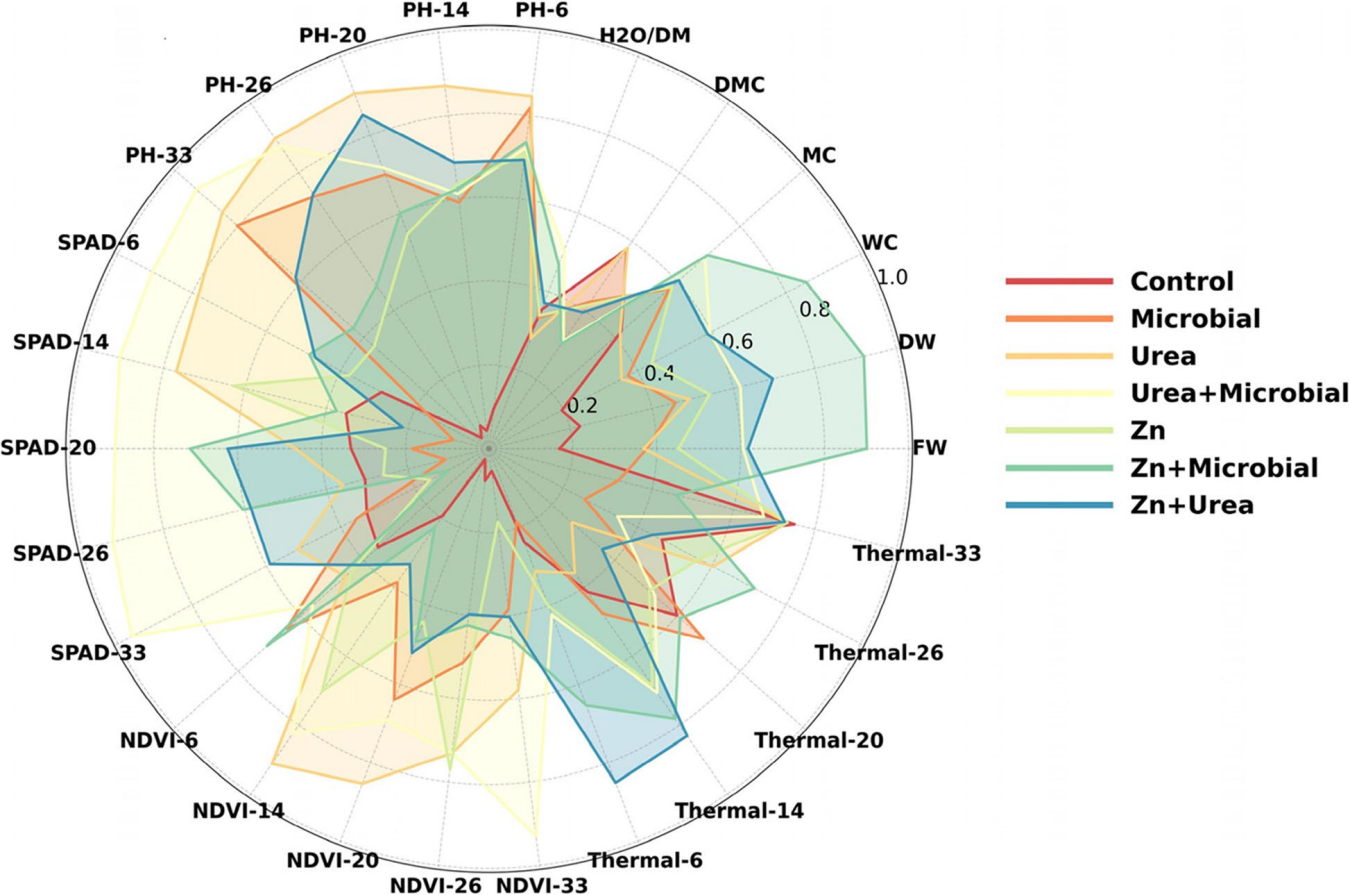
Radar diagram illustrating the effects of different fertilizer applications (Control, Microbial, Urea, Urea + Microbial, Zn, Zn + Microbial, Zn + Urea) on physiological, morphological, thermal, and biomass traits of soybean. Larger polygons indicate higher values across parameters, while smaller polygons represent lower performance, allowing direct comparison of treatments

When the radar diagram is examined, it becomes clear that the Zn + Urea and Zn + Microbial treatments form the broadest polygons, indicating superior overall performance. These combinations were particularly prominent in plant height (PH), SPAD, NDVI, and thermal parameters, reflecting enhanced photosynthetic capacity and improved stress regulation. They also exhibited higher values in terms of water relations (WC, MC, H₂O/DM) and biomass indicators (FW, DW) compared to the control group and single applications. Urea alone showed noticeable improvements in certain physiological parameters, while microbial fertilizer alone contributed more moderately, acting in a supportive role. The control group consistently formed the smallest polygon, representing the lowest performance across all measured traits. Taken together, these findings highlight that the combined application of zinc and urea, either directly or in integration with microbial inoculants, enhances the overall physiological performance of soybean and positively contributes to yield potential (Fig. 5).

**Fig. 6:**
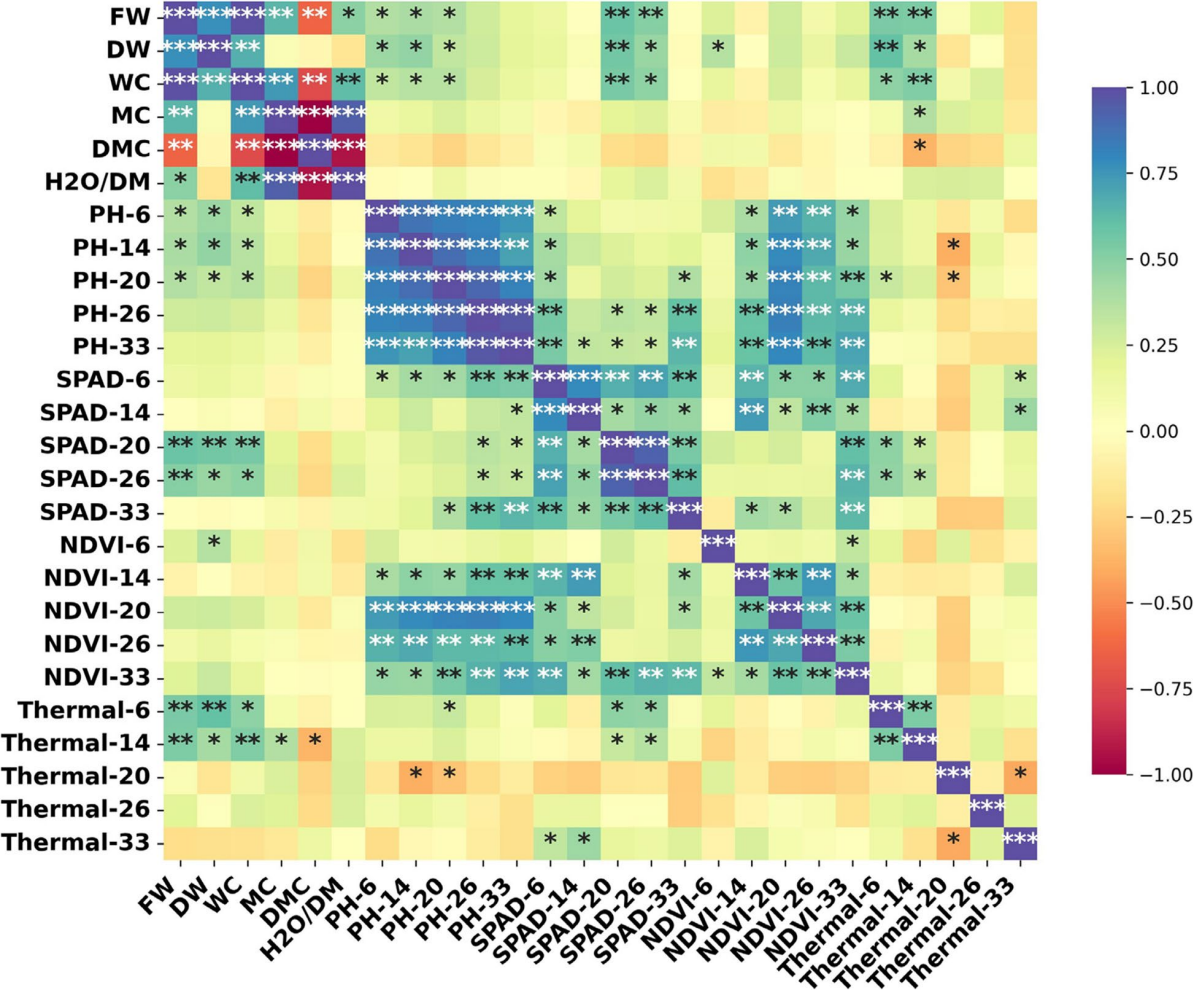
Correlation heatmap showing the relationships among physiological (SPAD, NDVI, canopy temperature), morphological (PH), and biomass-related traits (FW, DW) in soybean. Positive correlations are indicated in blue and negative correlations in red, with significance levels represented by asterisks (*p < 0.05; **p < 0.01; ***p < *0.001)*

Correlation analysis revealed strong linear relationships among the measured parameters (Fig. 6). SPAD and NDVI were highly and positively correlated (*r* > 0.80, *p *< 0.01), indicating that both indices reliably reflected chlorophyll status and photosynthetic capacity. Plant height (PH) also showed significant positive correlations with SPAD, NDVI, and biomass traits (FW and DW), demonstrating that enhanced vegetative growth was closely linked to improved physiological performance. In contrast, canopy temperature was negatively correlated with SPAD, NDVI, and PH (*r* < –0.60, *p* < 0.05), suggesting that cooler canopy profiles were associated with greater photosynthetic efficiency and higher biomass accumulation. These results emphasize the integrative role of optical indices in capturing plant growth dynamics and demonstrate that thermal imaging provides complementary information by detecting stress-induced changes in canopy temperature.

**Fig. 7:**
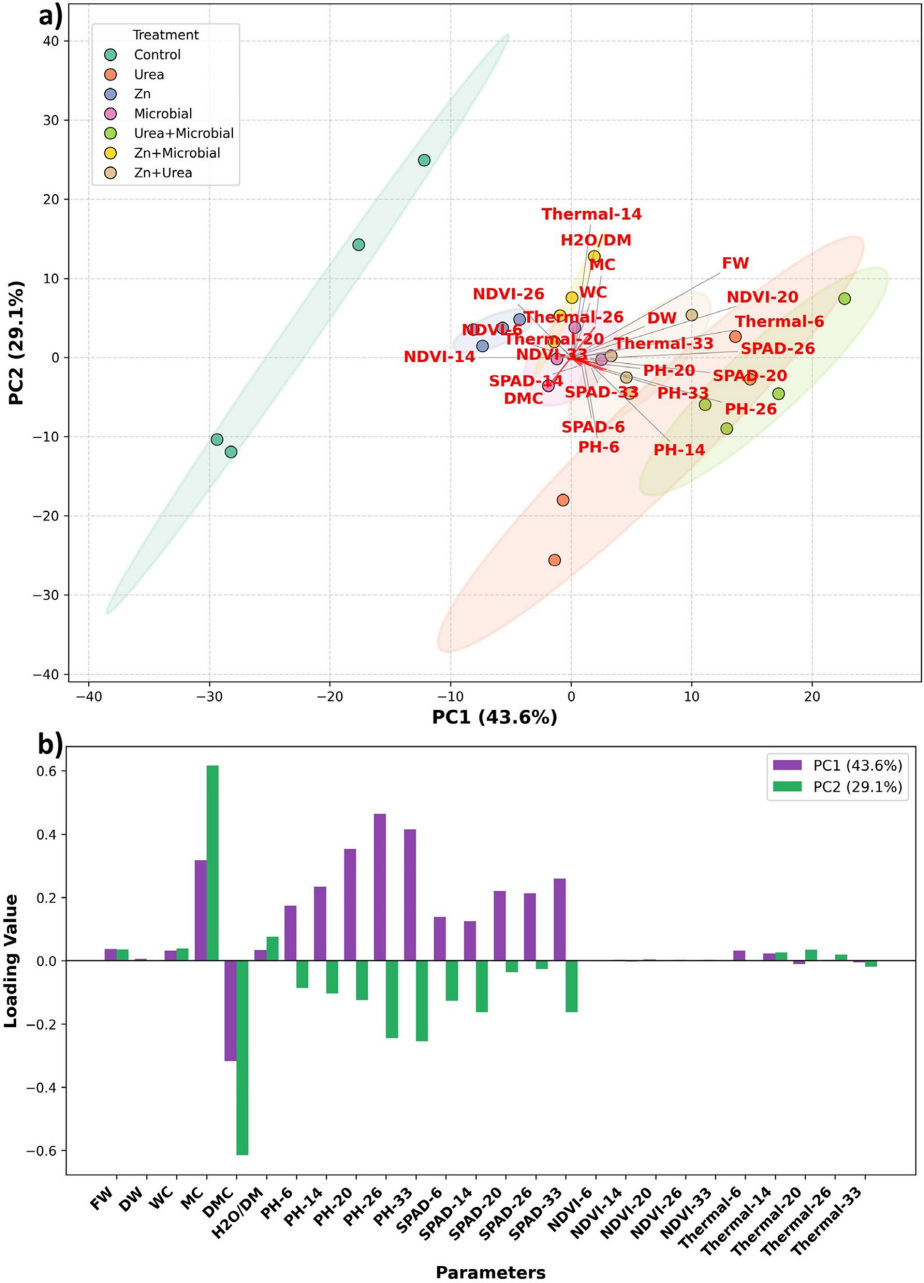
Principal Component Analysis (PCA) of measured physiological and biochemical traits under different fertilization treatments. **a** PCA biplot illustrating the distribution of treatments along the first two principal components (PC1 and PC2). Treatment groups are color‐coded, and ellipses represent group dispersion. Red labels denote the measured variables included in the analysis. **b** Loading plot showing the contribution of each parameter to PC1 and PC2. Positive and negative values indicate the direction of variable loadings within the PCA space

Principal component analysis (PCA) provided an integrated overview of the relationships among treatments and measured traits (Fig. 7). The first two components explained a large proportion of the total variance, clearly separating combined fertilizer treatments from the control and single applications. Urea + Microbial and Zn + Microbial treatments were positioned in close association with SPAD, NDVI, and plant height vectors, indicating their strong influence on photosynthetic activity and vegetative growth. Zn + Urea clustered near the biomass traits (FW and DW), highlighting its contribution to dry matter accumulation. In contrast, the control group was located in the opposite direction of these vectors, reflecting its overall weak performance. Canopy temperature loaded negatively on PC1 and was positioned away from SPAD and NDVI, confirming the inverse relationship between thermal stress and photosynthetic performance. These results demonstrate that PCA effectively summarizes treatment responses and highlights the superior physiological and agronomic performance of combined fertilizer applications compared to single treatments.

## Discussion

### Findings of optical sensor

SPAD values increased markedly during the early growth stages and clearly distinguished between treatments. Urea and Zn applications enhanced leaf chlorophyll content, reflecting their roles in chlorophyll biosynthesis and photosynthetic regulation [25, 26]. Similarly, in common bean, combined organic and inorganic fertilizer applications significantly increased SPAD and GreenSeeker values, which were closely associated with yield improvement [45]. Microbial inoculants sustained SPAD at later stages, likely by improving nitrogen mineralization through root–rhizosphere interactions [46]. These patterns suggest that SPAD can function not only as a descriptive parameter but also as an actionable indicator in nutrient management. Previous research in soybean and wheat has shown that SPAD values falling below ~ 35–37 are strongly associated with yield loss due to N or Zn deficiency [47, 48]. In this context, SPAD can provide growers with a reliable and cost-effective proxy for timely fertilizer adjustments.

NDVI peaked during mid-growth, coinciding with maximum vegetative development. Urea promoted canopy density and leaf area index, while Zn and microbial treatments enhanced photosynthetic activity, resulting in higher NDVI compared to the control. These findings align with earlier reports linking NDVI to biomass and leaf area [33, 49]. However, NDVI saturation at later stages reflects a well-known limitation in dense canopies [50], suggesting that combining NDVI with thermal imaging may overcome this constraint. Threshold analysis is also promising: NDVI < 0.60 has been linked to nutrient stress in legumes [51], and our results indicate that such benchmarks could be applied to soybean for early detection of suboptimal growth. Importantly, recent research in wheat has also shown that NDVI and related indices (e.g., INSEY) are among the strongest predictors of yield when analyzed across growth stages, further underscoring their diagnostic value for stress detection and productivity forecasting [52].

Thermal imaging further highlighted treatment effects on plant stress. Urea and microbial treatments reduced canopy temperature, indicating greater stomatal conductance and transpiration, while control plants showed elevated temperatures under stress. The negative correlation between NDVI and canopy temperature confirms that denser, healthier canopies were cooler, consistent with previous studies [53]. Importantly, thermal imagery allowed visualization of stress before visible symptoms occurred, supporting its role as a non-destructive early-warning tool. When used alongside optical sensors, thermal indices increase diagnostic precision, which is particularly valuable for precision agriculture [54].

### Morphological and postharvest relationships

Plant height complemented sensor findings, with urea promoting the tallest plants and microbial treatments supporting balanced growth, while the control remained lowest. Although plant height alone is a weak predictor of yield, its positive associations with SPAD and NDVI reinforce its role as part of an integrated assessment [24]. Multivariate analysis (PCA) confirmed this integrative role: combined treatments (Urea + Microbial, Zn + Microbial, Zn + Urea) clustered with biomass- and chlorophyll-related vectors, while control treatments were separated, indicating limited physiological performance [29]. Biomass responses confirmed the physiological patterns: fresh and dry weights were enhanced by urea and Zn, with microbial inoculants contributing particularly to dry matter stability. These results reflect nitrogen’s role in protein synthesis and leaf expansion, and zinc’s role in enzymatic activity and root development [26]. Interestingly, microbial inoculants compensated for later-stage stress by sustaining dry matter accumulation, which may be linked to enhanced nutrient solubilization and water uptake [27]. This suggests that microbial applications not only improve resource efficiency but may also buffer plants against environmental variability. Correlation analyses provided additional insights: SPAD was strongly associated with dry weight, NDVI with fresh biomass, and canopy temperature negatively with physiological traits. Such complementary correlations validate the use of combined sensors for yield prediction, echoing findings in cereals where SPAD–NDVI integration significantly improved prediction accuracy [55]. Establishing crop-specific thresholds could further refine predictive models, facilitating transition from research to field-scale automation.

### Water dynamics and thermal responses

Water content (WC) increased notably under Zn + Microbial and Zn + Urea, while dry matter content (DMC) peaked with urea, reflecting nitrogen’s role in structural biomass. The H₂O/DM ratio was highest under microbial–mineral combinations, consistent with improved water uptake and stomatal regulation [26, 46]. These results underscore the critical interaction between nutrient supply and water relations: by enhancing osmotic balance and transpiration efficiency, Zn and microbial inoculants contributed to stress resilience. Thermal imagery supported these findings, as high WC and H₂O/DM values corresponded with lower canopy temperatures, indicating efficient water-use strategies. Such integration of water and thermal traits is increasingly emphasized in climate-resilient agriculture [56].

### Contributions and limitations

This study contributes by integrating optical, thermal, morphological, and biomass traits into a holistic framework for soybeans. Unlike many studies focused solely on macronutrients (N, P, K), this work addresses micronutrient stress (Zn) and biological inoculants, offering novel insights into underexplored dimensions of plant nutrition [48]. Strong correlations between SPAD/NDVI and biomass reinforce their predictive reliability, while canopy temperature emerges as an early stress diagnostic. By identifying tentative thresholds for SPAD (~ 35) and NDVI (~ 0.60), the study bridges sensor data with practical agronomic benchmarks, which could be embedded into digital farming platforms and decision-support tools. Limitations include the controlled single-season design, restricted to the vegetative phase, which reduces generalizability. UAV-based systems could provide broader coverage and higher temporal resolution. Moreover, the absence of yield and quality traits (e.g., protein, oil content) is a limitation, as these are critical for linking physiological responses to end-use value [29]. Future studies should expand across locations, seasons, and varieties, validating sensor thresholds under variable field conditions. Such efforts would enable the transition from pot-based findings to scalable precision farming solutions.

## Conclusion

This study demonstrated that the combined use of optical sensors (SPAD, NDVI), morphological traits (plant height), thermal imaging, and post-harvest biomass parameters provides a robust and holistic framework for monitoring soybean growth under different fertilization regimes. SPAD and NDVI proved to be reliable indicators of chlorophyll content and canopy vigor, while canopy temperature emerged as a sensitive stress diagnostic, complementing biomass- and height-based assessments. The integration of microbial inoculants with urea and zinc consistently enhanced physiological performance, water-use efficiency, and biomass allocation, highlighting the agronomic value of combined nutrient strategies. Importantly, the identification of potential threshold values for SPAD (~ 35) and NDVI (~ 0.60) offers practical benchmarks for nutrient and stress management, bridging physiological monitoring with on-farm decision-making and automated fertilization systems. The close relationships observed between sensor-based indices and biomass accumulation further validate the role of non-destructive sensing in predicting crop performance. Moreover, the integration of water balance traits (WC, DMC, H₂O/DM) with thermal imagery provided novel evidence of how fertilization strategies shape water-use efficiency and stress resilience.

Despite these contributions, the study was limited to controlled conditions, a single season, and the vegetative stage. Future research should extend sensor-based monitoring to multi-season, multi-location field trials and include final yield and quality traits, while scaling the approach with UAV-based platforms to improve spatial and temporal coverage. Such efforts will be critical to validate sensor thresholds under diverse environments and ensure applicability in real-world farming systems.

Overall, the findings provide new insights into soybean responses to integrated nutrient management and confirm the utility of optical and thermal sensing as practical tools in precision agriculture. Incorporating these approaches into digital farming platforms has the potential to optimize nutrient strategies, improve water-use efficiency, and enhance the sustainability of crop production.

## Data Availability

The data will be available from the corresponding author on reasonable requests.
